# Correlation of safety behavior, handover quality, and risk perception: A cross-sectional study among Chinese psychiatric nurses

**DOI:** 10.3389/fpsyt.2022.1043553

**Published:** 2022-12-19

**Authors:** Yakun Liu, Weiyu Teng, Chen Chen, Guiyuan Zou

**Affiliations:** ^1^Department of Healthcare Respiratory, Shandong Provincial Hospital Affiliated to Shandong First Medical University, Jinan, Shandong, China; ^2^Department of Psychiatric, Shandong Mental Health Center, Shandong University, Jinan, Shandong, China

**Keywords:** handover quality, risk perception, safety behavior, psychiatric nurses, mediation

## Abstract

**Background:**

Nurses’ safety behaviors played an important role in patients’ safety goal realization, and it varies from person to person. However, less research has explored the safety behavior level of psychiatric nurses and its influencing factors. Thus, this research aimed to assess the level of safety behavior and explore whether risk perception mediated the relationship between handover quality and safety behavior among psychiatric nurses.

**Methods:**

A total of 186 registered psychiatric nurses in a Chinese hospital were recruited for this study, through the convenience sampling method. Handover quality, risk perception, and safety behavior were measured. Hayes’ PROCESS macro was used to evaluate the mediation of risk perception between handover quality and safety behavior.

**Results:**

Scores of psychiatric nurses’ safety behaviors were (47.98 ± 7.45), and handover quality and risk perception could predict the variance of nurses’ safety behaviors. Risk perception could partially mediate between handover quality and nurses’ safety behaviors, and the value of the mediating effect was 49.17%.

**Conclusion:**

Psychiatric nurses’ safety behaviors have a large promotion space. Therefore, healthcare professionals should endeavor to improve the handover quality of psychiatric nurses and decrease their risk perception, thereby promoting nurses’ safety behaviors.

## Introduction

Patient safety is an essential parameter of healthcare quality and is characterized by the avoidance, prevention, and amelioration of unfriendly results or harms resulting from the process of medical services (1). Every point in the course of medical services contains a certain degree of intrinsic unsafety. It is reported that approximately 1 in 10 inpatients experience harm, with at least 50% being avoidable. All healthcare practitioners have a professional responsibility to preserve patient safety and prevent adverse events during the provision of care in healthcare settings. Studies have shown that probably 66.7–85% of patient safety problems occur due to employees’ unsafety behavior (1, 2). Nurses are key patient safety links of patients and other healthcare professionals and play a significant role in promoting patient safety and improving patient outcomes (3). Nurses’ safety behaviors refer to a range of performances that nurses take on the work to safeguard patients from harm or promote patient safety, which might be manifested in compliance with nursing work regulations, correct implementation of nursing procedures, effective execution of medical advice, objective recording of nursing documents, and management of hospital environment (4–6). Safety behavior is vital for nurses to ensure the patients’ safety goal realization. However, there are significant drawbacks in aspects of patient identification, medication safety, patient involvement, risk assessment, fall management, and interpersonal communication (7). Those receiving hospitalized care in psychiatric institutes face similar risks (e.g., medication errors, falls, and ulcers pressure) to patients in the general hospital. Moreover, some of the unsafe behaviors associated with serious psychiatric symptoms (e.g., hallucination and delusion), and the emergency measures (e.g., restraint and seclusion) taken to cope with these may result in more challenges to patient safety (8). However, the safety behavior level of psychiatric nurses and its influencing factors have not been reported until now.

Nursing handover is defined as a real-time process in which one nurse hands over the responsibility of care for hospitalized patients to another nurse and occurs two or three times a day in healthcare settings (9). Nursing handover, a critical connection in nursing service, provides not only an opportunity for nurses to exchange patient-specific information but also a platform for nurses to interact and communicate and obtain organizational support (9, 10). Efficient nursing handovers can strengthen their awareness of risk or emergency handling, deliver potential risk information of adverse events, avoid medical errors or safety hazards and regulate safe nursing behavior (11, 12). On the contrary, deficient or failed nursing handovers can have severe consequences for hospitalized patients by affecting nurses’ job performance. But less research has discovered the association between handover quality and psychiatric nurses’ safety behaviors.

Nurses’ safety behaviors vary from person to person. Some authors found that safety behavior results from human–environmental factors concerning sociodemographic characteristics, safety culture, and organizational support (13–16). Moreover, prior research found that positive risk perception can promote individuals to choose and adopt nursing safety behaviors, thereby improving the quality of patient care (17, 18). Risk perception, characterized as an individual’s subjective judgments about the likelihood of various objective risks, has been a research focus in the field of risk management (19, 20). Information on risk interacts with knowledge, personal values, and personal beliefs to produce a subjective expression, that is, perception. Behaviorists suggested that proper risk perception helps to increase individual risk awareness and supports them to actively control the relevant risk factors (21). For example, Oyapero et al. found that nursing students with positive perceptions displayed more hand hygiene behaviors in clinical settings (22). Sellick et al. found that nurses who participated in risk management improvement projects usually suffered few needlestick injuries (23). Thus, we can summarize that proper risk perception was accompanied by positive work behavior and increased risk perception was expected to be associated with reduced unsafety behaviors. However, few studies explored the associations between safety behavior and risk perception among psychiatry nurses.

Within the context of the growing impact of heavy workloads, frequent shifts, and higher bed turnover rates, identifying the factors behind the formation of risk perceptions becomes an increasingly important task. Some research indicated that enormous individual and social-level factors are related to risk perception and risk perception as a mediator influences individuals’ job performance or psychosomatic health (24, 25). Having a better perception of the risks in advance can help individuals to implement preventive measures, reducing adverse event incidence rates in the hospital environment. Sand-Jecklin reported that handover quality is one of the effective ways to ensure medical safety (26). Thus, handover is a key risk point. Handover quality was highly relevant to risk perception, the better the handover quality, the more positive the risk perception (27). Furthermore, nurses’ perceptions of a potential threat sometimes do not cohere with the real situation and can come from misinformation. Information accuracy is one of the most important connotations of handover quality. Therefore, we speculated that risk perception might act as an intermediary mechanism underlying the relationship between handover quality and psychiatric nurses’ safety behaviors. As far as we know, no published studies have verified this hypothesis.

The cross-sectional research aimed to realize the following goals: (1) to investigate the level of psychiatric nurses’ safety behaviors; (2) to examine the associations between handover quality, risk perception, and safety behaviors; and (3) to explore the mediating effect of risk perception between handover quality and psychiatric nurses’ safety behaviors.

## Materials and methods

### Participants and procedure

This descriptive cross-sectional research was approved by the Ethics Committee of Shandong Mental Health Center and was performed in December 2019. Using convenience sampling, a total of 206 psychiatric nurses were recruited in a tertiary psychiatric hospital in Shandong Province, China. The inclusion criteria were as follows: (1) age between 18 and 60 years; (2) employment duration of >1 year; (3) a registered nurse with a Chinese Nurse Practitioner Certificate, providing face-to-face care for hospitalized patients in psychiatry or psychology department; and (4) those who were voluntary and anonymous to participate in the study. The exclusion criteria were as follows: (1) age <18 or >60 years; (2) employment duration of <1 year; (3) informal employee of the ward including practice or visiting nurses; (4) those on leave for >3 months; and (5) those who were reluctant to participate in research.

Sampling and data collection were performed completely online. First, the principal investigator contacted the head nurse of each ward to get a list of nurses and invited them to act as supervisors and facilitators of the study. The self-rated online questionnaire was created with Survey Star,^1^ and the online recruitment documents embraced the presentation of the researchers, the purposes or informed consent of research, the time to complete the survey, and the formal self-rated questionnaires. Second, the principal investigator distributed the questionnaire link to each head nurse, who then distributed the link to all nurses in their department. Third, all participants provided informed consent electronically prior to the formal self-reported questionnaires. The informed consent page presented two options (yes/no). Only participants who chose yes were taken to the questionnaire page, and subjects could quit the process at any time. Finally, of the 206 participants, 186 (90.29%) completed all questions on the survey.

### Participants’ sociodemographic characteristics

Basic sociodemographic characteristics consisted of age, gender (male or female), education level (bachelor’s degree or above or junior school or under), marital status (single or married), professional title (junior title or intermediate title or senior title), year of working, and job type (temporary nurse or permanent nurse).

### Nurse safety behavior questionnaire

The original Nurse Safety Behavior Questionnaire consisted of 12 items (established by Shih) and was widely used to assess nurses’ safety behaviors (4). Each item was rated on a 5-point Likert from 1 (hardly) to 5 (always). Answers were added up to obtain the overall scores, ranging from 12 to 60, with a higher score suggesting a higher level of nurses’ safety behaviors. The Chinese version demonstrated higher reliability and validation (28, 29), and Cronbach’s α in our research was 0.87.

### Handover evaluation scale

Handover quality was measured using the 13-item Handover Evaluation Scale developed by O’Connell (30). Each item was rated on a 7-point scale ranging from 1 (do not agree at all) to 7 (agree completely). Items were summed, with higher scores implying a better handover quality. A study on a Chinese nursing sample reported satisfactory validity and reliability (31), whereas Cronbach’s α for this study was 0.96.

### Nurse risk perception questionnaire

Risk perception was assessed *via* the Chinese version of Nurse Risk Perception Questionnaire (32), a 28-item evaluation measure. The Nurse Risk Perception Questionnaire originated from Zhang’s study, which was formatted by using qualitative interviews and the expert inquiry method (32). Each item was rated on a 1 (hardly) to 5 (almost always) scale. Thus, higher scores indicated more sense of professional risk factors. The Nurse Risk Perception Questionnaire showed good internal consistency and validity (33, 34), and Cronbach’s α in this survey was 0.93.

### Statistical analysis

Data analysis was performed using IBM SPSS 22.0 and the PROCESS macro version 3.2 (35, 36). Descriptive statistics were used to describe and compare the general characteristics of the participants. Pearson’s correlation analysis was used to determine the relations between variables. Hierarchical multiple regression analysis and the bootstrap method analysis were used to verify the mediation hypothesis. For all analyses, a *p*-value of < 0.05 was considered statistically significant.

## Results

### Description of sociodemographic characteristics and distribution of nurses’ safety behaviors in categorical items

As shown in Table 1, more than two-thirds of the participants were aged between 31 and 45 years. The participants were found to be women (77.4%), married (87.6%), a nurse for >10 years (61.3%), a permanent nurse (83.3%), have an intermediate (49.5%) or junior title (43.5%) and have a bachelor’s or higher degree (96.8%). Significant differences were found between nurses’ safety behaviors and the year of working (*p* < 0.05) and professional title (*p* < 0.001).

**TABLE 1:** Sociodemographic information and distribution of nurse safety behavior in categorical items (*N* = 186).

Variables	*n* (%)	Nurse safety behavior ($\bar{\chi }$ ± S)	t/F	*P*
Sex			1.509	0.221
Male	42 (22.6)	46.67 ± 7.25		
Female	144 (77.4)	48.36 ± 7.49		
Age			1.359	0.260
≤30 years	37 (19.9)	46.70 ± 7.12		
31∼45 years	126 (67.7)	47.99 ± 7.37		
>45 years	23 (12.4)	49.96 ± 8.27		
Marital status			0.388	0.534
Single	23 (12.4)	45.43 ± 6.16		
Married	163 (87.6)	48.34 ± 7.56		
Education level			0.060	0.807
Bachelor’s degree or above	180 (96.8)	48.27 ± 7.32		
Junior school or under	6 (3.2)	39.33 ± 6.38		
Employment duration			3.560	0.015
1∼3 years	6 (3.2)	43.33 ± 3.67		
3∼5 years	15 (8.1)	43.87 ± 4.78		
5∼10 years	51 (27.4)	47.16 ± 6.66		
>10 years	114(61.3)	49.13 ± 7.92		
Professional title			11.736	<0.000
Junior title	81 (43.5)	45.83 ± 7.11		
Intermediate title	92 (49.5)	48.82 ± 7.36		
Senior title	13(7.0)	55.46 ± 3.29		
Job type			2.835	0.094
Temporary nurse	31 (16.7)	46.52 ± 5.13		
Permanent nurse	155 (83.3)	48.27 ± 7.81		

### Scores and correlations among handover quality, risk perception, and nurses’ safety behaviors

Descriptive results for the quality of handover quality, risk perception, and nurses’ safety behaviors are depicted in Table 2. Participants’ average scores of nurses’ safety behaviors, handover quality, and risk perception were (47.98 ± 7.45), (76.10 ± 14.78), and (77.42 ± 15.23) respectively. The results of the correlations among the key variables (Table 2) showed that risk perception was negatively related to nurses’ safety behaviors and handover quality (*r* = −0.492, *p* < 0.001; *r* = −0.483, *p* < 0.001), whereas handover quality was significantly positively related to nurses’ safety behaviors (*r* = 0.379, *p* < 0.001).

**TABLE 2:** Mean, standard deviations (SD), and correlation of variables (*r*).

Variables	$\bar{\chi }$ ± S	Nurse safety behavior	Risk perception	Handover quality
Nurse safety behavior	47.98 ± 7.45	1		
Risk perception	77.42 ± 15.23	−0.492*	1	
Handover quality	76.10 ± 14.78	0.379*	−0.483*	1

*P < 0.001.

### Mediating effect of risk perception among different variables

**FIGURE 1:**
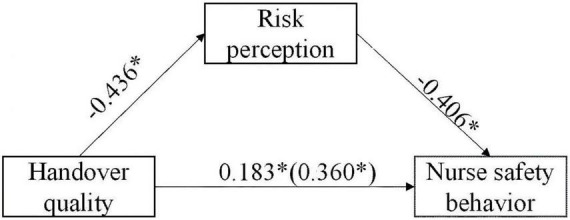
Mediation model of risk perception on handover quality and nurse safety behavior *P < 0.001.

Table 3 presents the results of the multiple regression analysis for nurses’ safety behaviors. The results show that the control variable (professional title) was significantly associated with nurses’ safety behaviors in step 1 (*R*^2^ = 0.105). Handover quality was also a significant positive predictor of nurses’ safety behaviors in step 2, explaining a 12.4% variance in nurses’ safety behaviors. In step 3, risk perception negatively predicted nurses’ safety behaviors, accounting for an 11.9% variance in nurses’ safety behaviors. When risk perception was added to the regression model, the regression coefficients for handover quality (from β = 0.360 to β = 0.183, *p* < 0.01) were reduced. Following the procedures of Baron and Kenny’s meditation test, (36) we can infer that risk perception partially mediates between handover quality and psychiatric nurses’ safety behaviors. Results of the bootstrapping method (Table 4) revealed that the path coefficient of the indirect effect of handover quality on psychiatric nurses’ safety behaviors was 0.0893 (95% CI: 0.0535, 0.1356), supporting the assertion that risk perception is a partial mediator (Figure 1). The indirect effect of handover quality on the nurses’ safety behaviors through risk perception accounted for 49.17% of the total effect. Together, handover quality, risk perception, and sociodemographic characteristics explained a 34.8% variance in nurses’ safety behaviors.

**TABLE 3:** Hierarchical linear regression analysis results.

Variable	Nurse safety behavior		
	
	Step 1 (β)	Step 2 (β)	Step 3 (β)
**Block 1**			
Years of working	0.051	0.131	0.222**
Professional title	0.291**	0.194*	0.086
**Block 2**			
Handover quality		0.360*	0.183**
**Block 3**			
Risk perception			−0.406**
*F*	10.787**	18.012**	24.166**
*R* ^2^	0.105	0.229	0.348
*ΔR^2^*	0.105	0.124	0.119

*P < 0.05 and **P < 0.01.

**TABLE 4:** Results for the direct and indirect effects of handover quality on nurse safety behavior with risk perception as mediator.

Mediation model hypothesis	Effects	Point estimate	95% CI	
			
			LL	UL
Handover quality- Risk perception- Nurse safety behavior	Total effect	0.1816	0.1152	0.2479
	Direct effect	0.0922	0.0238	0.1606
	Indirect effect	0.0893	0.0535	0.1334

## Discussion

The study first investigated the correlates of psychiatric nurses’ safety behaviors and explored the relations between handover quality, risk perception, and psychiatric nurses’ safety behaviors. It is also the first study focusing on risk perception’s mediation between handover quality and nurses’ safety behaviors. The details were as follows: scores of psychiatric nurses’ safety behaviors were above the medium level; safety behavior was associated with the year of working and professional title; handover quality and risk perception were significant positive predictors of safety behavior; and risk perception played a partial mediating effect between handover quality and psychiatric nurses’ safety behaviors.

Nurses’ safety behaviors as a modifiable protective factor affecting patient safety have received increasing attention. Prior surveys have uncovered the association between nurses’ safety behaviors and the incidence of adverse events (11, 12, 37). This survey shows that the score of psychiatric nurses’ safety behaviors is (47.98 ± 7.45), indicating that psychiatric nurses’ safety behaviors are above the medium level. However, scores of psychiatric nurses’ safety behaviors were significantly lower than that of nurses from general hospitals, suggesting that it leaves much space to improve for psychiatric nurses. A possible explanation is that the formation of psychiatric hospital safety culture lags behind that of general hospitals, and psychiatric nurses received less level of organizational support or safety culture course training (33, 37). Our study revealed a significant association between professional title, years of working, and nurses’ safety behaviors, which is consistent with Chu et al.’s findings (29). Chu et al. found that those nurses with low seniority or junior title usually lack clinical work experience, have limited ability to identify, foresee, or manage risks, and have poor communication skills, thereby resulting in poor safety behavior (29).

Consistent with previous research showing that handover quality was closely related to the quality of nursing, patient safety, and patient satisfaction, the results of this study found that handover quality is also a vital predictor of psychiatric nurses’ safety behavior (11, 12, 38). By itself, handover quality explained 12.4% of the variance in nurses’ safety behaviors. Handovers are instrumental for care continuity as evidence shows that they are a source for nurses’ job performance, especially in psychiatry (11, 12). Meanwhile, an abundance of research has indicated the beneficial effects of nursing handover quality improvement programs in patient safety (39, 40). High quality of handover is integral to cultivating a nursing safety culture, enhancing nurses’ safety awareness and participation in the modern healthcare setting. In contrast, lower quality of handover implies the defects or interruptions of nurses’ behavior. Therefore, it is necessary to acknowledge that a quality improvement program for handover is important for psychiatric nurses.

This survey reported that risk perception was negatively correlated with psychiatric nurses’ safety behaviors, the lower risk perception, the more nurses’ safety behaviors, which were observed in an existing research survey (41–43). Risk perception is an important predictor of safety behavior (41). Several studies in the field of occupational safety have revealed that risk perception was highly relevant to occupational safety precautions, such as the usage of hearing protection devices (41) and involvement in safety management (43). Meanwhile, nurses’ risk perception is a negative predictor of risk response behavior. Nurses with good risk perception adopt more positive and defensive coping behavior in the face of various professional risk factors. On the contrary, nurses with a poor risk perception are more susceptible to various professional risk factors and perceive the practice environment as risky; these circumstances subsequently increase the risk of psychological problems or burnout. Consequently, nurses with a poor risk perception usually strive to manage the negative effects by the avoidance or surrender coping style. Considering the destructive effects of risk perception on nurses’ safety behaviors, it would appear that interventions that focus on reducing risk perception, such as professional training or sessions involving risk management, should be suggested.

The most outstanding finding in this survey was the risk perception’s mediation between handover quality and psychiatric nurses’ safety behaviors, which suggest that handover quality not only directly influenced nurses’ safety behaviors but also indirectly influenced nurses’ safety behaviors *via* risk perception. Prior research found an association between handover quality and risk perception; in particular, handover is beneficial to nurses, both in terms of risk perception (27). Handover is a high-risk point in the continuity of care, given that it is used to deliver patient-specific information and risk factors and formulate robust care plans. Nurses with a better risk perception are more inclined to accept information about warnings in the handover process, as confirmed by earlier research (44–46). When the perceived risk is high, nurses tend to adopt active coping and self-protection behaviors. The indirect effects of handover quality on nurses’ safety behaviors through risk perception accounted for 49.17% of the total effects, suggesting that risk perception may play a major role in the development of psychiatric nurses’ safety behaviors.

This study has some limitations. First, as the study is cross-sectional, any causality interpretations between variables should be made with caution. Future longitudinal research should emphasize risk perception on both its antecedents and consequences. Second, the data were self-reported and collected online in the study. These could affect the reliability of the research. A final limitation of the study is the fact that the participants come exclusively from a single psychiatric institution; the results, therefore, will be verified with a further extension of the sample to wider areas of the country.

## Conclusion and practical implications

Better handover quality and proper risk perception of psychiatry nurses undoubtedly affect their professional performance as well as patient safety. Many studies suggested the effectiveness of quality improvement projects in improving the nursing handover process (40, 47). These findings indicate that good handover is a skill that can, and should, be taught and developed. Consequently, we suggest that handover theory and, in particular, skills training should be introduced into the field of psychiatry nursing to arm nurses with the confidence and competence to perform appropriate clinical handover. This study indicated that most psychiatric nurses were concerned about their professional risks. Empirical evidence supported that risk perception stems from social and cultural factors (48, 49). These findings highlight the need for hospital administrators’ efforts to improve physical and psychosocial working conditions and create a safe working environment for psychiatric nurses. Therefore, healthcare managers should endeavor to improve the handover quality of psychiatric nurses and decrease their risk perception, thereby promoting nurses’ safety behaviors.

## Data availability statement

The raw data supporting the conclusions of this article will be made available by the authors, without undue reservation.

## Ethics statement

This study was reviewed and approved by the Ethics Committee of Shandong Mental Health Center. The patients/participants provided their written informed consent to participate in this study.

## Author contributions

YL contributed to the data analysis, interpreted the findings, and wrote the original draft. WT and CC conceived the study and contributed to project administration and manuscript review. GZ contributed to research design, project administration, data analysis, methodology, and manuscript writing and review. All authors read and approved the final manuscript.

## Footnotes

1
https://www.wjx.cn
